# Assembly rules for protein networks derived from phylogenetic-statistical analysis of whole genomes

**DOI:** 10.1186/1471-2148-7-S1-S16

**Published:** 2007-02-08

**Authors:** Mark Pagel, Andrew Meade, Daniel Scott

**Affiliations:** 1 School of Biological Sciences, University of Reading, Reading RG6 6AJ, UK

## Abstract

**Background:**

We report an analysis of a protein network of functionally linked proteins, identified from a phylogenetic statistical analysis of complete eukaryotic genomes. Phylogenetic methods identify pairs of proteins that co-evolve on a phylogenetic tree, and have been shown to have a high probability of correctly identifying known functional links.

**Results:**

The eukaryotic correlated evolution network we derive displays the familiar power law scaling of connectivity. We introduce the use of explicit phylogenetic methods to reconstruct the ancestral presence or absence of proteins at the interior nodes of a phylogeny of eukaryote species. We find that the connectivity distribution of proteins at the point they arise on the tree and join the network follows a power law, as does the connectivity distribution of proteins at the time they are lost from the network. Proteins resident in the network acquire connections over time, but we find no evidence that 'preferential attachment' – the phenomenon of newly acquired connections in the network being more likely to be made to proteins with large numbers of connections – influences the network structure. We derive a 'variable rate of attachment' model in which proteins vary in their propensity to form network interactions independently of how many connections they have or of the total number of connections in the network, and show how this model can produce apparent power-law scaling without preferential attachment.

**Conclusion:**

A few simple rules can explain the topological structure and evolutionary changes to protein-interaction networks: most change is concentrated in satellite proteins of low connectivity and small phenotypic effect, and proteins differ in their propensity to form attachments. Given these rules of assembly, power law scaled networks naturally emerge from simple principles of selection, yielding protein interaction networks that retain a high-degree of robustness on short time scales and evolvability on longer evolutionary time scales.

## Background

Protein interaction networks can be thought of as the phenotypes of sets of functionally linked genes. Organismal phenotypes emerge from the interactions among these network phenotypes and the processes they define. In this light, protein interaction networks hold out a promise of developing a network-based view of development, and of integrating proteomics into systems biology.

The defining feature of a network is the set of the links it describes among a group of interactors or nodes. The degree or connectivity *k *of a node in the network is the number of other nodes to which it connects. One of the chief ways to characterize a network is to record the number of connections each node makes, and plot its distribution. If the nodes in the network make connections to each other at random and with a fixed probability, then a statistically homogenous structure arises in which the probability that a node is connected to *k *other nodes is proportional to *p*(*k*) = *e*^-*λk*^, where *λ *describes the rate at which the probability of forming an additional attachment declines with k. As the description implies, in a homogenously scaled network, nodes on average connect to a similar number of other nodes ≈ 1/*λ*. A curious feature of many naturally occurring networks is that they systematically depart from random connectivity, *p*(*k*) instead being described by the relationship *p*(*k*) ∝ *k*^-*λ*^, where again *λ *is a characteristic of the network. Networks with this form of connectivity distribution are described as scale-free or power-law scaled. They differ from exponential networks in having a broad or 'fat' tail – that is, the number of nodes with a large number of connections is more than expected under a random attachment model.

**Figure 1:**
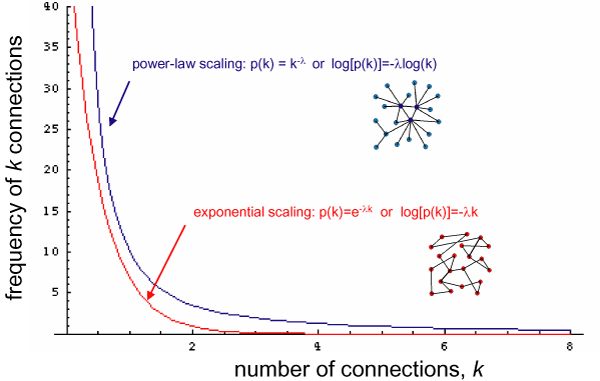
**Exponential and power-law curves**. Illustration of exponential and power-law curves, showing how power-law generates a larger number of highly connected nodes. Two network diagrams illustrate hypothetical differences in network connectivity expected for random (exponential) and scale-free networks.

Figure 1 illustrates exponential and power-law scaled relationships. Networks with power-law connectivity are inhomogeneous, their structure being dominated by a number of 'satellite' nodes each with a small number of connections, and a few 'hub' nodes with very large degrees of connectivity. Their description as 'scale-free' arises from the fact that, when measured on logarithmic axes, power-law scaled networks show a linear relationship, over a very wide range of connectivity, between the frequency of nodes with connectivity *k *and *k *itself. Power-law scaling has been documented for social networks [1], disease transmission networks [2], the distribution of links to World Wide Web pages [3], and even for citations to scientific papers [4]. In biology, protein interaction and metabolic networks also typically follow power-law scaling [5,6].

Scale-free topology does not emerge from the fixed-probability random attachment process and so various authors (e.g. [7]) have proposed a non-random attachment mechanism known as 'preferential attachment' or 'rich get richer' as a model for the growth of power-law scaled networks. Preferential attachment refers to a mechanism in which new interactions in a network are more likely to be made with nodes that already have a large number of connections. Scale-free networks emerge under preferential attachment if the probability of a new attachment being made to node *i *in a growing network is proportional to *k*_*i*_*/k *where *k*_*i *_is the number of existing connections to node *i*, and *k *is the total number of connections in the network.

Preferential attachment is a plausible mechanism for many kinds of network. In a social network, for example, a gregarious individual (high degree of connectivity) is more likely to be known to new individuals entering the network. For the same reasons, a new web page is more likely to link to well-known web pages, and highly cited papers are disproportionately likely to attract more citations. It is less clear how preferential attachment might arise in biological networks of proteins, requiring a protein to become more acquisitive of attachments the more it has.

One non-random mechanism by which networks grow is gene-duplication. When a gene duplicates the duplicated gene is assumed to acquire the original gene's connections, thereby increasing the overall connectivity in the network. Over time, duplicated genes tend to diverge and most of the original connections are lost. Simulation studies [8] suggest that gene-duplication followed by evolution of gene connectivity can lead to power-law scaling in networks. However Wagner [6] analysed the connectivity patterns of actual gene duplicates, and found that too many of the duplicated genes' connections are lost to influence the structure of the network. Instead, Wagner [6] reports phenomenological evidence among pairs of paralogous genes consistent with preferential attachment.

Do power-law scaled networks arise because of some selective advantage they confer or do they emerge from other more fundamental processes? Power-law scaled networks are more resistant to random loss of nodes than homogeneously connected networks [9], because most nodes have few connections, the majority of nodes being connected to one another via a small number of highly connected hub nodes. Owing to their small numbers, hub nodes are not likely to be affected by random removal (perhaps corresponding to a mutational loss in a biological network) and thus the topology of the network is relatively unchanged by random loss of nodes. By comparison, random removal of nodes in a homogeneously connected network has larger effects. This robustness of scale-free networks to random loss may translate into phenotypic stability in biological networks. On the other hand, Wagner [6] argues that power-law scaling simply emerges naturally from 'local rules', these rules being that there is a continual turnover of connections and nodes within networks over time, and that new connections are formed according to the principle of 'preferential attachment'.

Protein networks are evolving systems, meaning that questions about how they form and change over time can be studied directly on phylogenies. Recently we developed an approach for detecting pairs of functionally linked proteins from their pattern of co-evolution on a phylogenetic tree [10]. The method identifies independent instances of the evolutionary gain or loss of pairs of proteins. Applying the method to a data set of known functional links in the yeast [11], Barker and Pagel [10] identified 609 pairs of proteins that co-evolved in a sample of fifteen fully-sequenced eukaryotes, and a further 278 known functionally linked pairs that were found in every one of the fifteen species. The correlated gain/loss method substantially improved upon the conventional method of 'phylogenetic profiling' that merely seeks a correlation in the presence or absence of pairs of genes among a set of species (e.g. [12]).

Here we study questions about how protein networks evolve, using the 887 pairs of proteins we identified in our earlier study [10] as being functionally linked. The protein pairs can be used to infer a 'correlated evolution network' comprising 1774 pair wise functional links. We have information on the presence and absence in the sample of eukaryote species of the individual proteins that comprise the correlated evolution network. By applying phylogenetic methods of ancestral state reconstruction, we can reconstruct the probable points of origin on the eukaryote phylogeny of the various proteins. We can then ask at each node of the phylogeny what set of genes was present, and use this information to determine how many of the other proteins that a given protein is linked to were also present when that protein joined the network. Similarly we can calculate how many connections are removed when a protein is lost from the network. This allows us to build up a picture of where in the network – in the hubs or in the less connected proteins – most of the evolutionary turnover takes place. We can test directly for evidence that genes 'burrow in' to networks over time by acquiring additional attachments, and whether the patterns of acquisition of links conform to preferential attachment as the network evolves. Taken together, the patterns of gain and loss of proteins and their attachments, and the nature or topology of the attachments describes a set of 'rules of assembly' for evolving protein networks, and it is these that we wish to identify here.

## Results

### Phylogenetic tree

**Figure 2:**
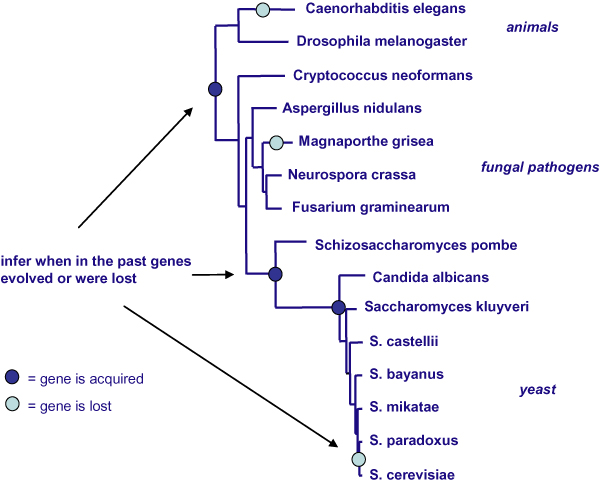
**Phylogenetic tree of eukaryote species**. Phylogenetic tree of fifteen eukaryote species used in this study (after Barker and Pagel, [10]). Dark blue dots illustrate hypothetical instances of a gene evolving and its protein entering the network; light blue dots illustrate instances of a gene and its protein being lost from the network.

Figure 2 displays the phylogenetic tree for the fifteen species we included in this study (tree drawn after [10]). The sample is biased towards yeast and fungal pathogens, representing the fully sequenced and well annotated eukaryotic genomes available at the time of our earlier study [10].

### The network and its scaling

**Figure 3:**
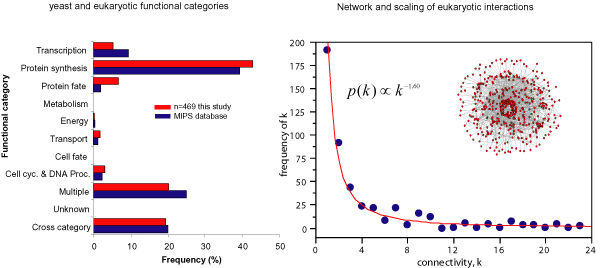
**Protein functional categories and protein network**. Left panel. A comparison of the proportion of yeast genes (from MIPS database) in various functional categories and the 469 genes in the eukaryotic correlated evolution network. The two sets of proportions are highly correlated (*r *= 0.95), but proportions do differ (*χ*^2 ^= 33.8, p < 0.001). Right panel. The correlated evolution network describing 1774 A<=>B connections among the 469 genes, and its connectivity distribution well described by a power-law (r^2 ^= 0.98).

We used the 469 proteins and the 887 pairwise functional links Barker and Pagel [10] identified in their sample of fifteen eukaryotes to investigate the correlated evolution network (Methods, data available from MP). The network defines 887 edges or 1774 connections and is drawn in Figure 3. The left panel records the proportion of yeast proteins in the MIPS database [11] assigned to various functional categories (blue bars) and for comparison the proportion in the same functional categories as found in our sample of 469 proteins. The two sets of proportions are highly correlated (r = 0.95, p < 0.0001) although there are differences among some categories (*χ*^2 ^= 33.80, 10 df, p < 0.001). More generally, there are few proteins associated with metabolism, energy, or cell fate, reflecting MIPS' emphasis on identifying actual physical protein complexes.

The average connectivity for a protein is 3.78 ± 4.47 (mean ± standard deviation) connections to other proteins. However, the network connectivity distribution is highly skewed (right panel) such that approximately 60% of the proteins have a connectivity of 1 or 2. The distribution is well characterized by a power-law, as has been reported for the yeast protein-interaction network (e.g. [6,11,13]). The fitted power-law relationship estimates *λ *to be 1.6 and predicts 98% of the variance in connectivity frequencies.

### Gains and Losses

Figure 2 illustrates how we can identify positions on the tree where a protein is gained and where it might later be lost. These ancestral state reconstructions provide a way to study turnover in the protein network. At the point a protein is gained, we calculate how many other proteins it is linked to that were also present in the network at that time. This is a protein's connectivity upon joining the network. A similar analysis calculates the connectivity of protein when it is lost from the network.

**Figure 4:**
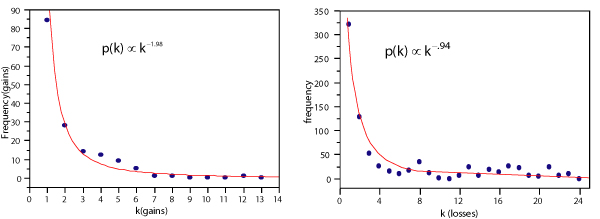
**Connectivity distributions for gains and losses of proteins**. Left panel: The connectivity distribution for genes at the time they enter the network. Based upon 174 genes that evolved after the root of the tree, and accounting for 395 new connections in the network (mean = 1.91 ± 1.77). Curve accounts for 95% of the variance in connectivity frequencies. Right panel: The connectivity distribution of genes at the time they were lost from the network. Curve accounts for 89% of the variance in frequencies. Based upon 239 genes comprising 847 losses or 3.54 ± 2.37 losses per protein (a protein can be lost in more than one place on the tree).

Figure 4 displays the connectivity distribution for proteins when they were gained (left panel) and for when they were lost (right panel) from the network. We identified 295 proteins present at the root of the tree, leaving 174 gained somewhere throughout the phylogeny. The 295 ancestral proteins have an average connectivity of 4.67 ± 5.26, accounting for 1379 links. The 174 acquired proteins account for 395 new connections or 1.91 ± 1.77 connections per protein upon entering the network. This is substantially lower than the average connectivity for the 295 ancestral proteins, suggesting that proteins that have been in the network for longer have more connections, possibly because they acquire connections over time. The connectivity distribution for gained proteins follows a power-law (*r*^2 ^= 0.95) and its exponent is somewhat steeper than that for the overall network.

Of the 469 proteins in the network, 239 were lost in at least one of the branches of the tree, with a mean of 3.54 ± 2.37 losses per protein or 847 losses in total. The average connectivity of proteins when lost from the network was 5.21 ± 6.48, higher than that for gained proteins, although the median connectivity is two. Fifty-nine of the 847 losses involved proteins that at the time of the loss had a connectivity of zero. The connectivity distribution for proteins with k > 0 when they are lost from the network is power-law scaled although with a shallower exponent than that for gains or for the network as a whole, and the long flat tail of the distribution shows why the average connectivity of lost genes is high.

Figure 4 shows that turnover in the protein-interaction network is dominated by proteins of low connectivity: 112 of the 174 new proteins (64%) had just one or two connections upon joining the network, and 53% (450/847) of the loss events from the network involved proteins with connectivities of 1 or 2.

### Preferential attachment

**Figure 5:**
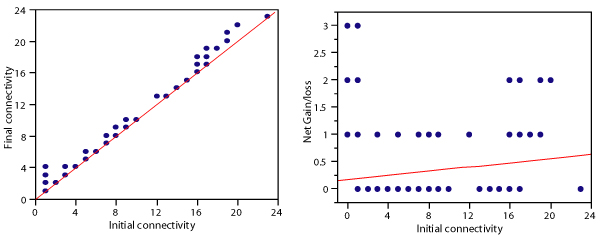
**Acquisition of protein links and test of preferential attachment**. Left panel. The relationship between the connectivity of a gene present at the root of the tree and its connectivity in the yeast (*S. cerevisiae*). Based upon 295 genes present at the root and in yeast. Line is 1:1 relationship. Right panel. The relationship between the number of connections acquired between the root of the tree and yeast (final-initial connectivity) and initial connectivity. Preferential attachment predicts a positive relationship. The regression line is positive but the weak relationship (*r*^2 ^= 0.03, slope = 0.018, p < 0.004), is not sufficient to produce a power-law scaled network (see text).

Preferential attachment predicts that genes with a larger number of links will acquire a greater proportion of the new links over time as the network evolves. We can test this directly from the ancestral presence/absence data. We recorded the connectivity of each of the 295 proteins reconstructed as present at the base of the tree, and then compared this to the number of links these proteins have in *S. cerevisiae*. Because all of the proteins in our data are present in *S. cerevisiae*, this path through the tree gives the greatest opportunity to detect preferential attachment. The left panel of Figure 5 plots the final connectivity versus the initial connectivity for these 295 proteins. The points all fall on or above the 1:1 line showing that the proteins all acquired links over the time this path through the eukaryote tree represents. Of the 295 proteins, 240 did not acquire any new connections. The remaining 55 proteins acquired 74 connections or a mean of 1.35 ± 0.58 connections per protein. The age of the animal/fungi divergence is controversial (see for example, [14]). However, adopting an age of approximately 1.5 × 10^9 ^years [15], the 74 newly acquired connections translates to approximately 1.6 × 10^-4 ^new connections per protein (n = 295) per million years, comparable to Wagner's [6] estimate of 5.9 × 10^-4 ^based on paralogous genes. The right panel of Figure 5 plots each protein's net number of gained links (final-initial) against its initial links. The greatest number of newly acquired connections occurred among the proteins with the fewest to start with – the opposite of that predicted by preferential attachment. The line shows the regression of gains onto initial connectivity (slope = 0.018), which although statistically significant, accounts for approximately 3% of the variation in newly acquired connections.

We conducted several simulation studies of an evolving network following Barabasi and Albert's [1] and Wagner's [6] model of preferential attachment, to determine whether the degree of preferential attachment we observe in Figure 5 could influence network structure. The preferential attachment model assumes that *p*(new attachment) ∝ , and generates power-law scaling in a growing network. We modified the preferential attachment rule to conform to the regression in Figure 5, making *p*(new attachment) = (0.17 + 0.018). This consistently returned randomly scaled (exponential) networks because the 0.17 intercept term dominates the probability of attachment.

### A variable rate of attachment model

A biologically plausible alternative to preferential attachment is to allow different proteins to have different fixed rates of attachment or 'stickiness'. We assume that this fixed rate of attachment influences the number of connections a protein makes when it enters the network and its likelihood of forming new connections. The variable rate of attachment model differs from the preferential attachment model in that the probability of forming a new attachment is independent of the number of current attachments a protein has or of the number of total network attachments. To motivate the model, let the probability that a protein forms *k *new links with other proteins be given by *p*(*k*) ∝ *e*^-*λk*^, where *λ *specifies the instantaneous rate of attachment. Applied to all proteins in a network, this is the random attachment model that returns homogenous network connectivity. However, consider that *λ *may vary from protein to protein such that some proteins are more and some are less likely to form links. This may be related to a protein's structure or to its function. Let *λ *have some probability density given by *f*(*λ*), then we can define the integral



The integral defines a new function *p*(*k*) describing the probability distribution of connectivity *k*, allowing for the rate of attachment to vary among proteins. Let *f*(*λ*) follow a gamma distribution such that



The gamma distribution is a very general probability density allowing for shapes ranging from an exponential (when *β *= 1) through to normal-like distributions depending upon the values of *α *and *β*.

Solving this integral yields

*p*(*k*) = (1 + *αk*)^-*β*^.

**Figure 6:**
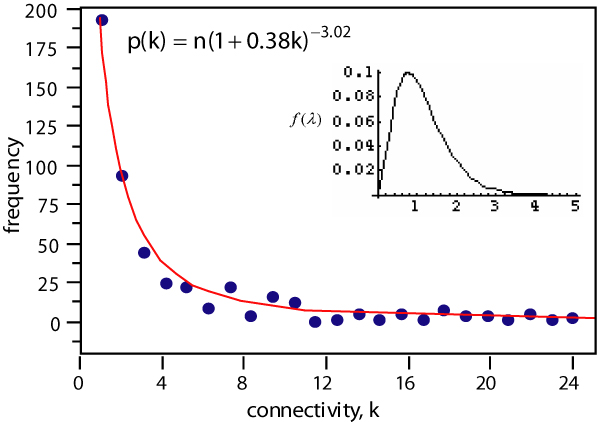
**The variable rate of attachment model**. Fit of the variable rate of attachment model (see text) fitted to the overall connectivity distribution of Figure 3 (right panel), accounting for 99% of the variance and illustrating that power-law scaling can emerge if proteins have different fixed propensities for forming attachments. Inset shows the predicted frequency distribution of attachment rates (*α *= 0.38, *β *= 3.02) under the variable rate of attachment model: most genes cluster around a low rate of attachment but a few show high rates of attachment, producing the 'hub' nodes in the network.

This equation for *p*(*k*) describes the connectivity distribution under a variable rate of attachment model. We fit this equation to the data of Figure 6, by choosing the values of *α *and *β *that minimized the sum of squared errors between the predicted and observed frequencies for each level of connectivity, k. This yields a curve (Figure 6) remarkably similar to that obtained from the power-law. The variable-rates model accounts for 99% of the variance in the connectivity frequencies, compared to 98% of the variance for the power-law curve.

Fitting the curve to the data yields estimates of *α *= 0.38 and *β *= 3.02. The inset to Figure 6 gives the shape of the estimated probability density *f*(*λ*) as given by these parameters. It is a right-skewed distribution with an expected mean value of *αβ *= 1.15 and an expected standard deviation of *αβ*^1/2 ^= 0.66. The interpretation is that most proteins have a low to medium rate of attachment but a few have high rates of attachment. By comparison, the simple power-law model fixes *λ *at 1.6.

The power-law and variable rate of attachment curves' fit to the connectivity frequencies can be compared by means of an F-test of their residual variances. Both curves require two parameters, a proportionality constant and an exponent for the power-law curve, and *α *and *β *for the variable-rates curve. The degrees of freedom for both residual variance estimates is therefore 20 (n-k-1), where n = 23 is the number of connectivity classes, and k = 2 is the number of parameters. This yields an F-ratio of F_20,20 _= 1.31, p ≈ 0.25.

## Discussion

We have studied a protein-interaction network based on pairs of proteins that have co-evolved in a sample of eukaryotic species. This eukaryotic 'correlated evolution' network differs from single species studies in identifying what might be a conserved set of eukaryote functional links. By reconstructing their presence or absence on a phylogeny of the eukaryotes we have been able to study how the network evolves.

We find that the correlated evolution network, like the interaction networks of single species has scale-free topology. Turnover in the interaction network is dominated by proteins of low connectivity: both newly acquired proteins and proteins lost from the network have power-law scaled connectivity distributions, meaning that most proteins that are gained or lost are connected to only a small number of other proteins. If connectivity in the protein interaction network is related to a protein's effect on the organism (e.g. [16-18]), then turnover in protein interaction networks may predominantly involve the so-called 'dispensable genes' [19] – those whose loss have only a negligible or even no measurable effect on the organism.

The biologist R.A. Fisher suggested in the 1930's [20] that genes would tend to evolve to have small effects. Fisher's argument was that if organisms occupy unimodal fitness landscapes, then small changes would be more likely than large ones to move them closer to the peak of the fitness distribution. Protein-interaction networks provide a modern perspective and a natural structure in which to interpret these suggestions. Fisher's genes of small effect may manifest themselves in the protein interaction network as genes of low connectivity. It is these that can be readily gained and lost owing to their small effects on the phenotype and fitness. About 80% of genes may be 'dispensable' [19], a number that is startling at first glance, but in fact expected if scale-free networks are the norm, and connectivity is related to effect size as supposed. We find, for example, that around 65% of the proteins gained or lost from the network had connectivities of two or less.

We did not find empirical support for the preferential attachment model (Figure 5) widely used to explain how power-law scaling arises [7]. If our results prove general, we suggest that the power-law curve, although providing a useful description of the data should not be assumed to confirm the model of preferential attachment. The variable-rates-of-attachment model we proposed in place of preferential attachment can also explain the typical 'fat-tailed' shape of the connectivity distribution of biological networks. It does so without invoking the requirement that a protein's propensity for acquiring attachments increases with the number of attachments it already has, and decreases as the number of attachments in the network rises ('preferential attachment'). Rather, the variable rates of attachment model asserts that some proteins intrinsically form attachments at higher rates than others, independently of their number of current attachments and of network connectivity as a whole. Even though this model fits the data only marginally better than a power-law curve, we prefer it on grounds of biological plausibility.

It may be difficult to distinguish the two models in practice. In a growing network, the variable-rate model would predict that proteins with more attachments might be more likely to acquire new attachments by virtue of having an intrinsically higher rate of forming such attachments. This is subtly different from preferential attachment but we suggest that the difference is biologically important. Thus, the variable rate of attachment model suggests that it should be possible to find and identify the features of proteins that determine their rates of attachment. For example, the proteins with high predicted rates of attachment under the variable rates model are expected to be the hub proteins in networks. The distribution of attachment rates predicts that there will be a relatively small number of these, and this is what is observed. By comparison, the peripheral proteins that form few attachments are predicted to have lower intrinsic attachment rates.

Whether scale-free networks are directly selected for being evolvable, error tolerant or robust, or whether the scale-free topology emerges out of more fundamental processes remains an open question. Fisher's principle of genes evolving to have small effects combined with the variable rate of attachment model suggests that scale-free topology naturally emerges from just two simple rules of assembly: proteins differ in their inherent capacity to form attachments to other proteins, and proteins with small effects will tend to be gained and lost more readily. There is no need to posit preferential attachment rules or selection for the scale-free topology.

Nevertheless scale-free networks and their rules of assembly may give insight into one of the key problems of development: how reliably to produce a complex phenotype in an unpredictable environment. In real time, organisms must develop and then maintain a highly complex phenotype, far more complex for example than computers, large buildings, or even the Space Shuttle – objects whose complexity in human terms makes them prone to sometimes catastrophic breakdown. Organisms must be able to achieve a stable phenotype reliably and repeatedly despite unpredictable environmental conditions. Selection may even act more strongly on variance-stabilizing mechanisms in development than on the mean value of traits [21]. Scale-free networks may have the relative independence from input conditions that has been observed in real biological systems (e.g. [22]), and which may be necessary to achieve a stable phenotype. Selection acting among variants of interaction networks (such as among the individuals in a population) may favour network structures that confer real-time stability [23,24]. This sort of robustness, arising from the topology of the network, in turn may confer evolvability on organisms over evolutionary time because the same network characteristics that make it possible to overcome environmental fluctuations in real time, give the organism an advantage in adapting at the genetic level to semi-permanent changes to the environment or to the demands of occupying a new niche.

This view along with the structure and phenotypic characteristics of protein-interaction networks invites speculation about fundamental questions regarding the origin and evolution of phenotypic diversity. Loosely, one can divide phenotypic diversity into two classes. In one, organisms are largely shape-transformations of one another, differing in size and life history but not in gross aspects of morphology and development. Many family-level clades may fit this description. The other class contrasts organisms that differ in important details of the body plan, such as fish and mammals. The question for a systems and network-based science of development is whether the evolutionary mechanisms underlying this variation differ qualitatively or quantitatively. That is, do typical family level differences arise from a gradual process of the accumulation of many changes of small effect, corresponding to the dominant turnover we observe in the protein interaction network? In contrast, do larger developmental differences arise from more rapid and almost saltationary changes corresponding to the infrequent, but nonetheless observed, acquisition or loss of a highly connected or hub proteins in the network? What might be caricatured as the 'Hox gene school of development' might favour the latter interpretation, whereas a more gradualist view would favour the former for both kinds of difference at the phenotypic level. This is a fundamental and as yet uninvestigated feature of development that can be studied with methods such as we have used here. The answers promise to integrate our understanding of development and the phenotype with the growing fields of proteomics and systems biology.

## Conclusion

A few simple rules can explain the topological structure and evolutionary changes to protein-interaction networks: most change is concentrated in satellite proteins of low connectivity and small phenotypic effect, and proteins differ in their propensity to form attachments. Given these rules of assembly, power law scaled networks naturally emerge from simple principles of selection, yielding protein interaction networks that retain a high-degree of robustness on short time scales and evolvability on longer evolutionary time scales.

## Methods

### Phylogenetic inference

The tree was inferred from EF1-*α *and EF2 gene-sequences obtained for the fifteen eukaryotic species in Figure 2 ([10] for details, see [25]).

### Gene presence/absence data

The Munich Information Centre for Protein Sequences [26,27] database of protein complexes lists 260 known *S. cerevisiae *protein complexes, the 1156 proteins that form them, and the functional categories into which they can be classified (including transcription, protein synthesis, metabolism, and energy). The MIPS functional links have been determined by low-throughput laboratory procedures and therefore provide a reliable collection of functional links in this species. Barker and Pagel [10] identified the presence/absence of each of these proteins in the fourteen other eukaryotic species, using a reciprocal best-in-genome global alignment between proteins.

### Identification of correlated functional links

Regarding each protein within a MIPS *S. cerevisiae *complex as functionally linked to every other different protein, Barker and Pagel [10] identified 5619 pairs of proteins that could be studied for correlated evolution across species. Correlated evolution is assessed using a procedure that compares the log-likelihood of a continuous-time Markov model of trait evolution in which the two proteins evolve independently of each other on the phylogeny, to that obtained when the two proteins' evolution is described by a model in which their evolution is correlated [10,28-30]. This procedure identified 609 or 11% of pair wise links as being significantly correlated across species.

In a further 278 pairs of proteins both members of the pair are found in all fifteen species we studied. We consider these pairs also to represent functional linkages as they are annotated as functionally linked in *S. cerevisiae *and have both been retained throughout the evolutionary history of the eukaryotes. Combining these two sets gives 887 edges in the network or 1774 total connections. We use this set here to construct the correlated evolution network.

### Ancestral state reconstruction

Four hundred and sixty nine proteins comprise the 1774 total links. For each of these proteins, we reconstructed its probable first appearance on the tree, and then later losses from the protein's pattern of presences and absences among the species. Ancestral presence/absence was determined by the methods described in Pagel, Meade, and Barker [25]. In particular, at each node of the tree we calculate the probability that the protein was present. Proteins are regarded as present at nodes when this probability is greater than or equal to 90%. This corresponds to a likelihood ratio in favour of the protein being present of ~2, following the criteria outlined in Pagel [31].

## Authors' contributions

MP conceived the project, designed the analyses, and wrote the manuscript. AM wrote the computer software for performing the analyses of correlated evolution and for reconstructing ancestral states [32] and conducted these analyses on the data set. DS wrote scripts for analysing protein networks links and drew the network diagrams. All authors approved the final draft of the manuscript.
